# Serum concentration of gastrin, cortisol and C-reactive protein in a group of Norwegian sled dogs during training and after endurance racing: a prospective cohort study

**DOI:** 10.1186/s13028-016-0204-9

**Published:** 2016-04-26

**Authors:** Marte Ekeland Fergestad, Tuva Holt Jahr, Randi I. Krontveit, Ellen Skancke

**Affiliations:** 1 Department of Companion Animal Clinical Sciences, Faculty of Veterinary Medicine and Biosciences, Norwegian University of Life Sciences, PO Box 8146 Dep, 0033 Oslo, Norway; 2 Department of Companion Animal Clinical Sciences, Faculty of Veterinary Medicine and Biosciences, Norwegian University of Life Sciences, PO Box 8146 Dep, 0033 Oslo, Norway; 3 Department for Pharmacoeconomics, Norwegian Medicines Agency, PO Box 63, Kalbakken, 0901 Oslo, Norway; 4 Department of Food Safety and Infection Biology, Faculty of Veterinary Medicine and Biosciences, Norwegian University of Life Sciences, PO Box 8146 Dep, 0033 Oslo, Norway

**Keywords:** Sled dog, Canine, Long distance race, Gastrin, Cortisol, C-reactive protein, Gastritis, Gastric ulceration

## Abstract

**Background:**

High incidences of gastritis and gastric ulceration are observed in sled dogs participating in endurance races. Exercise-induced increases in hormones like gastrin and cortisol have been suggested as possible contributing factors. An increase in C-reactive protein (CRP) has also been observed in canines during physical exercise. The aim of this study was to evaluate the effect of long distance racing on the serum concentration of gastrin, cortisol and CRP in a group of sled dogs, by comparing the results achieved early in the training season and after participating in a long distance race; “Femundløpet”. Dogs that only trained to the race, but did not compete in the race, were used as control dogs. Sixty-five sled dogs participated in the study; 46 competing dogs (25 completing and 21 non-completing the race) and 19 non-racing dogs (control dogs). The blood samples were collected in October 2012 and February 2013.

**Results:**

The post-race serum concentration of gastrin, cortisol and CRP was significantly elevated in sled dogs participating in the race (both completing and non-completing dogs) when compared to the results from training. However, no significant differences were observed between the two sampling dates in the control dogs. Post-race results for completing and non-completing dogs were also compared. This demonstrated a significant elevation in gastrin in non-completing versus completing dogs, and a significant elevation in cortisol in completing compared to non-completing dogs.

**Conclusions:**

Participation in a long distance race was associated with a significant increase in serum gastrin, cortisol and CRP in sled dogs.

**Electronic supplementary material:**

The online version of this article (doi:10.1186/s13028-016-0204-9) contains supplementary material, which is available to authorized users.

## Background

Physical activity can be associated with gastrointestinal dysfunction in both humans and dogs, although the pathophysiology is complex and not completely understood [1–5].

Sled dogs competing in endurance races have a high risk of gastric erosion, ulceration and bleeding [3, 4]. The prevalence of gastric lesions in dogs competing in the Iditarod race has been reported to be 48.5–61 % [3, 5]. By assessing necropsy findings of dogs deceased during or soon after competing in an endurance race, the two most common causes of death were found to be aspiration of gastric contents with subsequent aspiration pneumonia and acute blood loss due to gastric ulceration. Gastritis and gastric ulceration were common autopsy findings [4].

It has been suggested that gastritis and gastric ulcers in sled dogs, independent of the severity, often are asymptomatic, and that gastric lesions do not necessarily affect the performance of racing dogs [6]. However, in humans these conditions are related to pain and discomfort [7]. As such, their high prevalence in sled dogs has significant animal welfare implications. This highlights the importance of understanding the pathophysiology and identifying the potential causes of exercise-related gastritis and gastric ulcers.

Release of gastrointestinal and stress-related hormones, like gastrin and cortisol, has been proposed as potential contributors to exercise-induced gastritis and gastric ulcers in sled dogs [1, 6].

In humans, participation in long distance races leads to an increase in serum gastrin [8–10]. The major effect of gastrin is stimulation of gastric acid release, and gastrin is therefore assumed to play a role in all gastric inflammatory processes [11]. If a similar rise in gastrin also exists in racing sled dogs, a possible gastrin-initiated increase in gastric acid may contribute to exercise-induced gastritis and gastric ulcers.

Physical exercise promotes release of cortisol in both humans [8, 9] and dogs [1, 12, 13]. An association between gastric lesions and an increase in endogenous cortisol has been demonstrated in racing sled dogs. It has been suggested that a rise in cortisol during training may be involved in the development of gastritis and gastric ulcers seen in racing sled dogs [1].

A rise in serum concentration of C-reactive protein (CRP) generally is related to trauma, inflammation or infection. Several studies have demonstrated an increase in CRP related to physical exercise [14–16], mostly due to muscle damage [16]. However, an exercise-induced increase in CRP together with other markers of gastric lesions may also indicate gastric inflammation during physical activity.

The aim of this study was to evaluate the effect of racing on the serum concentration of gastrin, cortisol and CRP in a group of sled dogs by comparing the results achieved early in the training season and after participating in a long distance race; “Femundløpet”. Dogs only trained and not competing in the race, were used as control dogs.

## Methods

Study design: clinical prospective cohort study.

“Femundløpet” is a long distance race of 600 km, arranged in Røros, Norway. Four mushers registered for participation in “Femundløpet” in February 2013, were contacted in October 2012. All agreed to let their dogs participate, and later signed a consent regarding the study.

### Included dogs

In this study, a group of sixty-five sled dogs (n = 65) were observed during a training season from 2012 leading up the race in February 2013. The dogs were divided into three cohorts based on participation and outcome of the race: race completing (group 1) (n = 25), race non-completing (group 2) (n = 21) and non-competing/control dogs (group 3) (n = 19). Non-completing dogs were defined as dogs withdrawn from the race without considering the reason for the withdrawal, such as illness or other causes.

The dogs were classified by breed (Alaskan or Siberian husky), sex (male/female) and age (in years). Information regarding health, training and feeding routines during the training season was collected via questionnaires.

### Blood sample collection

Blood samples were collected early in the training season (October 2012); samples A, and at the end of the observation period, shortly after the race (February 2013); samples B. Samples from the three groups were further subdivided into A1 and B1 (race completing), A2 and B2 (race non-completing) and A3 and B3 (non-competing/control dogs). The training data of the determined parameters served as control for the post-race values.

Prior to collection of samples A, the dogs had been rested and fasted for a minimum of 12 h. These samples were collected at the mushers’ homes.

B1 samples were collected as soon as possible and within 1.5 h after the dogs crossed the finish line. The completing dogs were not fed prior to blood sampling at the finish line. B2 samples were collected at the checkpoints as soon as possible and within 8 h after the dogs were withdrawn from the race. Feeding prior to sampling in non-completing dogs was recorded at the checkpoints. Dogs that had not been fed prior to sample collection were categorized as unfed. There was no recording of feeding along the track. B3 Samples were collected at the mushers’ homes 2 weeks after the race. The dogs had been resting and not fed for a minimum of 12 h prior to sampling.

### Blood sampling and storage

Blood samples were obtained from the cephalic vein, and approximately 6 ml of whole blood were collected from each dog. Within 1 h the samples were centrifuged for 10 min, and serum was transferred to labelled serum tubes. Serum isolated after one centrifugation was reserved for gastrin analysis. Serum isolated after two centrifugations was reserved for CRP and cortisol analysis. The sampling procedure was the same for all blood samples.

Samples A1, A2, A3 and B3 were immediately transported in a cooler and frozen at −70 °C. Samples B1 were relocated to −20 °C within 1 h after sampling. Samples B2 were kept in a cooler, outdoors at approximately −5 °C, and transferred to −20 °C within 2 days after collection. After the race, samples B1 and B2 were kept frozen during transport back to the Norwegian School of Veterinary Science and transferred to −70 °C. All samples were stored at −70 °C prior to analysing in March 2013.

### Analysis of gastrin, cortisol and CRP

Analysis of serum gastrin was performed at Rikshospitalet University Hospital, Oslo. Measurement of serum gastrin was performed via GASTRIN (^125^ I) Radioimmunoassay Kit (MP Biomedicals, USA). This analysis is designed for human gastrin measurement, but is currently also applied for measurement of canine gastrin. The reference range for gastrin (10–40 pg/ml) used in this study is obtained from another laboratory, which uses the same method (Helen Evans, BSc Hons, Veterinary Services Operations Manager, Cambridge Specialist Laboratory Services, UK, personal communication).

Analysis of serum cortisol and CRP were performed at The Central Laboratory at the Norwegian University of Life Sciences. Serum cortisol was analysed using IMMULITE 2000 from Simens, both instruments and reagents. The cortisol analysis is a solid-phase, competitive chemiluminescent enzyme immunoassay (polyclonal rabbit anti-cortisol). Serum CRP was analysed by ADVIA 1800 from Siemens, Immunoturbidimetric assay (anti-human CRP antibody), reagents from Randox (RANDOX Laboratories Ltd., UK), and calibrator from Life Diagnostics, Inc USA: dog C-Reactive Protein (2250 mg/L). The laboratory’s own reference values were employed (cortisol 20–250 nmol/L and CRP 0–15 mg/L).

### Statistical analysis

Data was compiled in Microsoft^®^ Excel (Microsoft Corporation, 1 Microsoft Way, Redmond, WA 98052-7329, USA) and subsequently imported in Stata 12 (Stata Corporation, 4905 Lakeway Drive, College Station, TX 778445, USA), which was used for all statistical analyses.

Distribution of gastrin, cortisol and CRP was assessed by histograms. Formal tests for normality (Shapiro-Wilks) were also applied. One-way ANOVA or Wilcoxon–Mann–Whitney tests were applied to test for differences in serum gastrin, CRP and cortisol between the variables breed, sex, age and fasting status. Wilcoxon–Mann–Whitney tests were used to compare median values of gastrin, cortisol and CRP in sample A1 with sample B1, A2 with B2, and A3 with B3. In addition, sample A1 was compared to A2 and A3 for possible differences before the race applying Wilcoxon–Mann–Whitney tests. Boxplots for gastrin, cortisol and CRP by the subgroups completing, non-completing and controls were generated.

For CRP, Wilcoxon signed rank test was used to test whether the median CRP values of A samples as well as A1, A2 and A3 were significantly different from and higher than the upper reference limit for CRP (15 mg/L). Because a few dogs in the B2 group (non-completing) were fed prior to blood sampling, the B2 group were divided in sub-groups fed and not-fed and compared to the respective A2 Samples applying Wilcoxon–Mann–Whitney tests. *P* values  <0.05 were considered statistically significant.

To confirm results of the non-parametric testing, quantile regression with sample (A1, A2, A3, B1, B2, B3) as variable were performed for the three outcome variables gastrin, cortisol and CRP. Overall significance of the variable was tested using multiple Wald test. *P* values <0.05 were considered statistically significant. The Stata command lincom was used to generate contrasts among the different categories.

As sensitivity-analyses, statistical analyses excluding the whole team that withdrew during the race, were also performed.

## Results

A total of 65 dogs, 18 Siberian huskies (28 %) and 47 Alaskan huskies (72 %), were included in the study. There were 32 females (49 %) and 33 males (51 %) with a mean age of 3.9 years, ranging from 1 to 9 years. There were no significant age or sex differences in the serum concentrations of gastrin, cortisol and CRP. However, there was a significant breed difference in the serum concentration of CRP (*P* = 0.017) (Table 1).

**Table 1:** Summary statistics for the variables gastrin, cortisol and CRP according to breed, age and sex

Variable	N (%)/mean (range)	Gastrin (pg/ml)		Cortisol (nmol/L)		CRP (mg/L)	
		Mean (sd)/median	*P* value	Mean (sd)/median	*P* value	Mean (sd)/median	*P* value
Breed			0.173*		0.20*		0.017*
Alaska husky	47 (72 %)	18.9 (11.7)/15.8		61.2 (24.4)/58.0		27.9 (16.7)/24.8	
Siberian husky	18 (28 %)	15.6 (6.8)/12.9		64.7 (23.4)/59.5		15.6 (18.2)/10.9	
Age	3.9 (1–9)	18.0 (10.7)/15.0	0.71**	62.3 (24.0)/58	0.67**	24.4 (17.9)/18.8	0.85**
Sex			0.13*		0.99*		0.23*
Male	33 (1 %)	15.2 (4.9)/14.3		63.0 (27.5)/58.0		27.1 (19.1)/21.8	
Female	32 (49 %)	21.1 (14.0)/15.0		61.3 (20.1)/57		21.5 (16.3)/17.8	

Summary statistics for the variables serum gastrin, cortisol and C-reactive protein (CRP) according to breed, age and sex in a group of Norwegian sled dogs, *Wilcoxon–Mann–Whitney test, **ANOVA.

Forty-six dogs (71 %) participated in “Femundløpet” 2013, while 19 dogs (29 %) were included as post-race controls. Twenty-five dogs (54 %) completed the 600 km race in 3–4 days, while 21 dogs (46 %), including a complete team of 12 dogs, were dropped at different times during the race. The team that withdrew completed 500 km of the race.

Information regarding health, training and feeding routines during the training season was considered approximately equal for all dogs. At the time of blood sampling in October 2012 (A1, A2 and A3), all dogs were considered healthy based on physical examination by the authors, and none had clinical signs of gastrointestinal dysfunction according to the owners’ observations. This was also the case for the control dogs post-race in February 2013 (sample B3). Among the completing dogs (B1) no recording of possible illness, including signs of gastrointestinal dysfunction, was done by the authors. Non-completing dogs (B2) were withdrawn due to various reasons, like lameness, depression, anorexia and/or dehydration. However, some of them did not have any signs of illness, which was the case with several of the dogs in the team withdrawn at 500 km.

Neither dogs during training (A1, A2, A3) nor the controls post-race (B3) were fed prior to blood sampling. Except for seven (15 %) non-completing dogs none of the competing dogs (85 %) were fed.

Sample A1 was compared to A2 and A3 for possible differences in gastrin, cortisol and CRP. No significant differences were found between A1 and A2, nor between A1 and A3. Summary statistics for these samples are outlined in Table 2.

**Table 2:** Summary statistics for the variables serum gastrin, cortisol and CRP

Sample category	N	Mean (sd)	Median (range)	*P* value*
Gastrin (10–40 pg/ml)				
A1 completing dogs	25	18.1 (11.2)	15.0 (8.3–62.4)	*P* < 0.001
B1 completing dogs	25	41.6 (17.8)	36.6 (13.5–71.1)	
A2 non-completing dogs	21	17 (12.2)	15.2 (9.1–64.7)	*P* < 0.001
B2 non-completing dogs	21	68.7 (28.3)	69.8 (16.4–120.8)	
Fed A2 non-completing dogs	7	21.9 (19.2)	15.9 (9.1–64.7)	*P* = 0.013
Fed B2 non-completing dogs	7	63.1 (28.9)	75.3 (17.1–95.5)	
Not-fed A2 non-completing dogs	14	15.9 (6.6)	14.35 (9.3–30)	*P* < 0.001
Not-fed B2 non-completing dogs	14	71.5 (28.6)	69.8 (16.4–120.8)	
A3 control dogs	19	18.1 (8.5)	14.7 (8.8–40.4)	*P* = 0.7703
B3 control dogs	19	20.5 (12.6)	18.2 (8.3–57.9)	
Cortisol (20–250 nmol/L)				
A1 completing dogs	25	60.8 (26.9)	57.0 (27–141)	*P* < 0.001
B1 completing dogs	25	153.2 (60.9)	147.0 (74–323)	
A2 non-completing dogs	21	58.1 (18.2)	57 (33–94)	*P* = 0.001
B2 non-completing dogs	21	103.7 (54.2)	80.0 (46–224)	
A3 control dogs	19	68.8 (25.7)	63.5 (41–135)	*P* = 0.0752
B3 control dogs	19	57.6 (23.3)	52 (34–125)	
C-reactive protein (0–15 mg/L)				
A1 completing dogs	25	25.8 (15.9)	22.1 (1.2–55.3)	*P* < 0.001
B1 completing dogs	25	90.6 (49.9)	75.1 (26.9–191.7)	
A2 non-completing dogs	21	20.4 (20.4)	13.5 (1.8–78.9)	*P* < 0.001
B2 non-completing dogs	21	104.5 (52.2)	89.7 (22.3–220.2)	
A3 control dogs	19	27.2 (17.5)	23.4 (5.3–63.3)	*P* < 0.001
B3 control dogs	19	15.3 (35.2)	3.2 (0–156.9)	

**Fig. 1:**
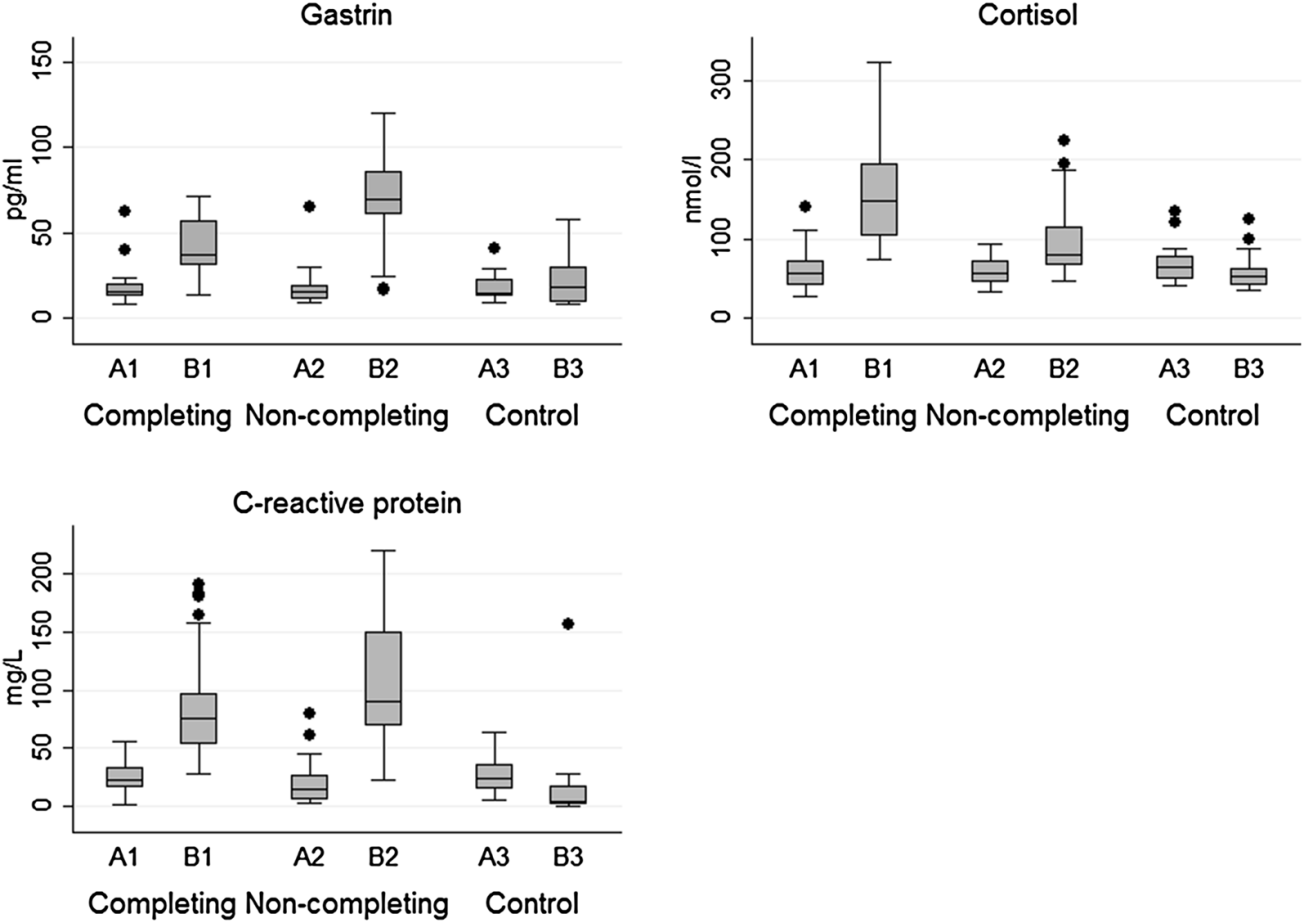
Boxplots for gastrin, cortisol and C-reactive protein. Boxplots for gastrin, cortisol and C-reactive protein [CRP] by the subgroups completing, non-completing and control dogs in a group of sled dogs participating in a Norwegian long distance race

Boxplot of the different subgroups are shown in Fig. 1. The following sections present the detailed results for gastrin, cortisol and CRP.

Summary statistics for the variables serum gastrin, cortisol and C-reactive protein (CRP) in a group of Norwegian sled dogs. Samples were taken early in the training season, after completing a long distance race, in dogs not completing the race and in non-competing control dogs. Reference values: gastrin 10–40 pg/ml, cortisol 20–250 nmol/L and CRP 0–15 mg/L. *Wilcoxon–Mann–Whitney test.

### Gastrin

The serum concentration of gastrin was significantly increased (*P* < 0.001) when comparing samples B1–A1 (race-completing dogs) (Table 2). This was also the case when comparing samples B2–A2 (race non-completing dogs) (*P* < 0.001). There was no significant difference in gastrin serum concentration between samples B3 and A3 (control dogs). Serum concentration of gastrin was significantly elevated in samples B2 (non-completing dogs) when compared to samples B1 (completing dogs), (*P* < 0.001).

When comparing the post-race results in the fed and not-fed dogs among the non-completing dogs, no significant difference in median serum gastrin concentrations was detected. In the fed non-completing dogs there was significantly higher median gastrin in the post-race samples compared to their respective A samples (*P* = 0.013) (Table 2). In the not-fed non-completing dogs, the post-race samples were also compared to their respective samples A. Also in these dogs the median gastrin was significantly higher post-race (*P* < 0.001) (Table 2). Sensitivity-analyses excluding the entire team that withdrew had negligible influence on the results (data not shown, available upon request).

Quantile regression analysis confirmed the results; all categories except from B2 (race non-completing) had significantly lower median serum gastrin compared to the baseline category which was the race completing group (B1) (Table 3), and group B2 had significantly higher gastrin than A2 (race non-completing dogs) (*P* < 0.001) while the difference between B3 and A3 (control dogs) was not significant.

**Table 3:** Quantile regression of serum gastrin, cortisol and C-reactive protein values

Variable and level	Coefficient (SE)	P value	95 % confidence interval
Gastrin			
Racing status		<0.001^a^	
A1 completing dogs	−21.6 (4.83)	<0.001^a^	−31.17; −12.03
B1 completing dogs	Baseline	–	–
A2 non-completing dogs	−21.4 (5.06)	<0.001^a^	−31.42; −11.38
B2 non-completing dogs	33.2 (5.06)	<0.001^a^	23.18; 43.22
A3 control dogs	−21.9 (5.20)	<0.001^a^	−32.20; −11.60
B3 control dogs	−18.40 (5.20)	<0.001^a^	−28.70; −8.10
Intercept	36.6 (3.42)	<0.001^a^	29.83; 43.37
Cortisol			
Racing status		<0.001^a^	
A1 completing dogs	−21.6 (4.83)	<0.001^a^	−31.17; −12.03
B1 completing dogs	Baseline	–	–
A2 non-completing dogs	−21.4 (5.06)	<0.001^a^	−31.42; −11.38
B2 non-completing dogs	33.2 (5.06)	<0.001^a^	23.18; 43.22
A3 control dogs	−21.9 (5.20)	<0.001^a^	−32.20; −11.60
B3 control dogs	−18.40 (5.20)	<0.001^a^	−28.70; −8.10
Intercept	36.6 (3.42)	<0.001^a^	29.83; 43.37
C-reactive protein (CRP)			
Racing status		<0.001^a^	
A1 completing dogs	−21.6 (4.83)	<0.001^a^	−31.17; −12.03
B1 completing dogs	Baseline	–	–
A2 non-completing dogs	−21.4 (5.06)	<0.001^a^	−31.42; −11.38
B2 non-completing dogs	33.2 (5.06)	<0.001^a^	23.18; 43.22
A3 control dogs	−21.9 (5.20)	<0.001^a^	−32.20; −11.60
B3 control dogs	−18.40 (5.20)	<0.001^a^	−28.70; −8.10
Intercept	36.6 (3.42)	<0.001^a^	29.83; 43.37

Results from quantile regression of the effect of racing status and time of blood sampling on serum gastrin, cortisol and C-reactive protein (CRP) in a group of sled dogs participating in a Norwegian long distance race

^a^Multiple wald test

The sensitivity-analyses excluding the entire team that withdrew from the race decreased the difference between group B2 compared to B1 (*P* = 0.094), but had negligible influence on the other results (data not shown, available upon request).

### Cortisol

The serum concentration of cortisol was significantly increased (*P* < 0.001) when comparing samples B1–A1 (race-completing dogs) (Table 2). This was also the case when comparing Samples B2–A2 (race non-completing dogs) (*P* = 0.001). There was no significant difference in cortisol serum concentration between Samples B3 and A3 (control dogs). Serum concentration of cortisol in post-race results was significantly higher (*P* = 0.0039) in completing dogs when compared to non-completing dogs. Sensitivity-analyses excluding the entire team that withdrew had negligible influence on the results (data not shown).

Quantile regression analysis confirmed the results; all categories had significantly lower median serum cortisol compared to the baseline category which was the race completing group (B1) (Table 3), while the differences between B2 and A2 (race non-completing dogs), and between B3 and A3 (control dogs) were not significant. The sensitivity-analyses excluding the entire team that withdrew increased the difference between B2 and A2 (race non-completing dogs) (*P* = 0.002), but had negligible influence on the other results (data not shown, available upon request).

### Crp

Median CRP for samples A was significantly higher than the upper reference value for CRP (*P* < 0.001). For sample A1 and A3, median CRP was also significantly higher than the upper reference limit, *P* = 0.004 and 0.02, respectively. For samples A2 the median CRP appeared to be only numerically higher than the upper reference limit.

The serum concentration of CRP was significantly increased (*P* < 0.001) when comparing samples B1–A1 (race completing dogs) (Table 2). This was also the case when comparing samples B2–A2 (race non-completing dogs) (*P* < 0.001). There was a significant increase in serum CRP concentration in samples A3 compared to B3 (control dogs) (*P* < 0.001). Serum concentration of CRP in post-race results was elevated in non-completing dogs when compared to completing dogs, although this was not significant. Sensitivity-analyses excluding the entire team that withdrew had negligible influence on the results (data not shown, available upon request).

Quantile regression analysis confirmed the results; all categories except B2 (race non-completing dogs) had significantly lower median CRP compared to the baseline category, which was the race-completing group (B1) (Table 3). Group B2 had significantly higher CRP than A2 (race non-completing dogs), (*P* < 0.001), while the difference between B3 and A3 (control dogs) was not significant. The sensitivity-analyses excluding the entire team that withdrew had negligible influence on the results (data not shown, available upon request).

### Quality control of gastrin, cortisol and CRP

MP Biomedicals inter-assay precision and intra-assay precision GASTRIN (^125 ^I) Radioimmunoassay Kit is outlined in Table 4.

**Table 4:** MP biomedicals inter-assay precision and intra-assay precision GASTRIN (^125^I) radioimmunoassay kit

Sample	Control 1	Control 2	Control 3	Serum
Inter-assay precision, gastrin				
n	20	20	20	20
Mean, pg/mL	56.7	232	465	49.4
S.D.	2.59	9.56	26.1	2.91
% CV	4.6	4.1	5.6	5.9
Intra-assay precision, gastrin				
n	22	22	22	
Mean, pg/mL	56.0	225	474	
S.D.	5.91	16.8	37.2	
% CV	10.6	7.5	7.8	

Regarding cortisol quality control, the inter-assay CV was 10 % at 80 nmol/L and 8 % at 550 nmol/L

Regarding CRP Quality Control, for homemade dog serum pool, the inter-assay CV was 6 % at 27.4 mg/L

## Discussion

Gastrin, cortisol and CRP are involved in different physiological mechanisms including secretion of gastric acid, stress response and inflammatory reactions. The main findings in this study were that dogs participating in this long distance race had an increase in serum concentration of gastrin, cortisol and CRP. It can be hypothesized that an increase in these parameters to some extent may indicate the general level of stress and inflammation arising during extreme physical exercise, but they may also be markers of gastrointestinal dysfunction.

### Gastrin

Sled dogs that participated in a long distance race experienced a significant increase in serum gastrin. Previous human studies, which focused on gastrointestinal dysfunction during long distance races, also concluded that there is an exercise-induced increase in serum concentration of gastrin [8–10, 17].

Exercise-induced elevation in serum gastrin can be a result of several factors or mechanisms. Repetitive blows against visceral organs during running are in bipeds thought to trigger the release of gastrointestinal hormones, including gastrin [8], although this has not been documented in quadrupeds. In addition, renal inactivation of gastrin might be reduced during physical exercise, due to redistribution of blood supply from visceral organs to skeletal muscles [8]. However, a study concluded that renal hypoperfusion was less likely in exercising sled dogs [18]. Release of catecholamines due to physical activity [17] may stimulate the release of gastrin, although a study regarding exercising sled dogs showed minimal systemic release of catecholamines, making this mechanism of gastrin increase less likely [12].

Food intake, along with the expectation and smell of food, stimulates secretion of gastrin [19]. In humans and horses, food intake after physical exercise gives a significantly higher increase in serum gastrin compared to what is registered in fasted individuals [17, 20]. Sled dogs are snacked during racing, and this may give a higher exercise-induced increase of gastrin, similar to the rise seen in humans and horses. In this study, all dogs were supposed to be fasted prior to blood sampling, however the competing dogs might have received food along the trail, and seven non-completing dogs were fed prior to blood sampling. It is also possible that competing dogs were expecting food when approaching the checkpoints and finish line. These factors may contribute to the increase in serum gastrin detected in the competing dogs. Serum gastrin was significantly elevated in non-completing dogs when compared to completing dogs. If this is a result of different feeding patterns or may be associated with illness in non-completing dogs is not known and merits further investigation.

The major pathophysiologic factor causing gastritis and gastric ulceration is currently considered to be breakdown of the mucosal barrier, and not hyperacidity as earlier proposed [21]. Some studies have concluded that there is no decrease in gastric pH in humans or dogs during physical activity [8, 9, 22–24], which emphasizes that hyperacidity is not the causation of exercise-related gastritis and gastric ulceration. Release of various inhibitors, such as somatostatin, has been assumed to limit the release of gastric acid during physical exercise [8, 9].

On the other hand, several studies regarding sled dogs demonstrate a significant reduction in the severity of exercise-induced gastritis and gastric ulceration by preventive use of gastric acid reducing agents, like omeprazole and famotidine [25–27]. This would suggest that gastric acid is a contributing factor to exercise-induced gastritis and gastric ulceration in dogs. Further investigation regarding exercise-induced increase in gastrin related to gastric pH should be done. Use of antacid medication, like omeprazole and famotidine, leads to an increase in serum gastrin concentration in dogs [28]. However, at the time of this study, use of such medications prior to and during race was not allowed in Norway. According to the mushers, none of the dogs were medicated prior to blood sampling during training. Therefore, use of antacids did not influence the gastrin results in this study.

### Cortisol

There was a significant increase in serum cortisol in the competing dogs after racing. These findings are in accordance with previous human, horse and canine studies [1, 8, 9, 13, 17, 29]. Also, anticipation of exercise before racing can cause increase in cortisol in sled dogs [30].

A negative energy balance during physical activity may trigger the release of cortisol [1, 12]. Racing sled dogs have high-energy requirements, and may not be capable of sufficiently increasing their food intake to meet these requirements. Racing sled dogs are also exposed to cold, which further increases energy demands [31].

Dogs that completed the race had significantly higher serum cortisol levels compared to dogs that did not complete. This may reflect the sustained strain that the dogs experience with greater distance, and the prolonged requirement for energy in completing dogs.

It has been proposed that exercise-induced increase in endogenous cortisol may contribute to development of gastritis and gastric ulceration in sled dogs. A study regarding sled dogs competing in the Iditarod race showed that dogs with abnormal gastroscopic findings after racing also had a significantly higher serum cortisol level, compared to dogs with normal gastroscopic findings. However, the association between endogenous cortisol and gastrointestinal ulceration was not possible to establish [1].

### Crp

There was a significant increase in serum CRP in competing dogs. An increase in serum CRP after long distance racing has previously been reported in humans and canines [14–16, 32]. Muscle degradation with subsequent inflammation is assumed to be a contributing factor in humans and dogs [16, 33].

Serum CRP concentration was slightly higher in non-completing dogs compared to completing dogs, although the difference was not significant. It could be speculated that this may be due to injuries or illness causing an inflammatory response in the majority of the non-completing dogs. Studies with larger case numbers and appropriate statistical power are necessary to reveal a true difference and support the proposed hypothesis.

The control dogs had a significantly higher serum concentration of CRP during training compared to the results post-race. It is likely that they underwent less physical activity in the post-race period compared to the training season. Also, median CRP for Samples A was significantly higher than the upper reference value for CRP, implicating either an inflammatory state of all dogs or a methodical problem in the laboratory analysis of CRP bringing higher results than previously described for healthy dogs when using the same assay. The serum concentration of CRP was significantly higher in Alaska huskies compared to Siberian huskies. The Siberian huskies in the present study represented a single team and one musher, and the finding might be spurious and possibly not reflect true breed differences.

In a study with experimentally induced damage to the gastric mucosa in dogs, it was concluded that CRP could be a useful indicator for gastric lesions [34]. However, as serum CRP also increases as a result of exercise or general illness in sleds dogs, the use of serum CRP as a general diagnostic tool for gastritis or gastric ulceration may be limited in racing sled dogs.

### Strengths and limitations

A strength of this study is the prospective design. The use of a control group for the post-race results and a genuine endurance race setting increase the external validity of this study. However, there were also limitations that need to be kept in mind.

Standardization of blood sampling during the race was difficult and not optimal. Appropriate storage of the blood samples was difficult to standardize completely, especially regarding the time passed before freezing at −70 °C. Although temperatures in February in this part of Norway are generally well below 0 °C, storage procedures could have influenced the results. It is known that serum gastrin concentration can decrease significantly if stored in room temperature for a few hours [35]. Likewise, the cortisol concentration can decrease if serum is stored at temperatures above 0 °C over several days [36]. CRP on the other hand is considered to be far more stable in different storage conditions, and it is likely that the serum concentration of CRP were not affected to the same extent as gastrin and cortisol, by transport and temperatures [37, 38]. It is therefore possible that an even greater rise in both serum gastrin and cortisol levels would have been measured, if the samples had been stored in optimal conditions.

Some samples from dogs that did not complete the race were collected after the dogs had been rested for up to 8 h. This could influence the values of the measured parameters. The impact of this resting period is probably a decrease in serum concentrations of gastrin, cortisol and CRP. It is possible that the measured values would have been even higher if the samples had been collected straight after racing.

Because the dogs were from different mushers, confounding factors in the dogs’ home environment might influence the results and interpretation of the statistical analysis.

One musher and his team of dogs withdrew from the race after 500 km. All dogs belonging to this team were recorded as non-completing. Having a whole team of dogs not completing the race was not preplanned during the design of the study, but merely a result of the study being performed in a genuine race setting. Sensitivity-analyses excluding this team had negligible influence on the results and conclusions.

During a race non-completing dogs mostly withdraw due to clinical signs of disease. However, the presumption that they are clinically ill and that completing dogs are healthy, is not always correct. In the authors’ experience, reasons for dropping may vary from observed clinical disease to consideration of a dog’s fitness. Withdrawal of entire teams, including sick and healthy individuals, also happens. There may be different opinions regarding when it is unjustifiable to let a dog continue a race, making the definition sick relative and subjective. Completing dogs have additionally been observed with signs compatible with lethargy and diarrhea. Consequently, the dogs’ health status should not be defined as healthy just because they have completed a race. These challenges need to be addressed in future studies.

No age or sex related differences in the serum concentrations of gastrin, cortisol or CRP could be detected in this study. This might be due to the sample size, and therefore could have appeared if the number of dogs included had been higher. However, it seems unlikely that there should be age or sex differences in either of these parameters. It can be speculated that a rise in CRP may appear with increasing age due to processes linked to ageing, or that a rise in cortisol may be observed in younger, inexperienced dogs due to a higher degree of psychological stress.

In the analysis of gastrin, both inter- and intra assay variation were available (Table 4), which strengthens the results of this study. CV was low and relatively similar for both inter- and intra-assay variation. In the analysis of cortisol and CRP, only inter-assay variation was measured. How this could have affected the results in this study is unknown.

### Future research

Further investigation is needed to evaluate if an exercise-induced increase in serum gastrin and cortisol may contribute to gastritis and gastric ulceration in racing sled dogs. In future studies it would be interesting to focus on the association between distinct signs of illness and different biomarkers in completing and non-completing racing dogs. However, the clinical evaluation of the participating sled dogs needs to be done by the same person in a standardized matter, and clinical suspicion of gastrointestinal disease needs to be confirmed by diagnostic procedures. Because sled dogs with gastritis and gastric ulcers often are asymptomatic [7], gastroscopy together with measurement of biomarkers should be performed during training season, and immediately before and after participation in a race. This could increase our knowledge concerning exercise-induced metabolic changes in sled dogs during racing and enlighten possible causalities to gastritis and gastric ulceration in racing sled dogs.

## Conclusions

This study indicated an exercise-induced increase in serum gastrin, cortisol and CRP in sled dogs participating in a long distance race. The results of this study have generated new knowledge on exercise-induced metabolic changes and can guide future research in this field (Additional file 1).

## Additional file

**Additional file 1:** Dataset of gastrin, cortisol and CRP. The excel file contains the complete dataset of all variables used in the different analyses of serum gastrin, cortisol and C-reactive protein (CRP) in a group of Norwegian sled dogs.

## References

[1] Royer CM, Willard M, Williamson K, Steiner JM, Williams DA, David M (2005). Exercise stress, intestinal permeability and gastric ulceration in racing Alaskan sled dogs. Equine Comp Exerc Physiol.

[2] Moses FM (1993). Gastrointestinal bleeding and the athlete. Am J Gastroenterol.

[3] Davis MS, Willard MD, Nelson SL, Mandsager RE, McKiernan BS, Mansell JK (2003). Prevalence of gastric lesions in racing Alaskan sled dogs. J Vet Intern Med.

[4] Dennis MM, Nelson SN, Cantor GH, Mosier DA, Blake JE, Basaraba RJ (2008). Assessment of necropsy findings in sled dogs that died during Iditarod Trail sled dog races: 23 cases (1994-2006). J Am Vet Med Assoc.

[5] Davis MS, Willard MD, Williamson KK, Steiner JM, Williams DA (2005). Sustained strenuous exercise increases intestinal permeability in racing Alaskan sled dogs. J Vet Intern Med.

[6] Ritchey JW, Davis MS, Breshears MA, Willard MD, Williamson KK, Royer CM (2011). Gastritis in Alaskan racing sled dogs. J Comp Pathol.

[7] de Oliveira EP, Burini RC (2009). The impact of physical exercise on the gastrointestinal tract. Curr Opin Clin Nutr Metab Care.

[8] O’Connor AM, Johnston CF, Buchanan KD, Boreham C, Trinick TR, Riddoch CJ (1995). Circulating gastrointestinal hormone changes in marathon running. Int J Sports Med.

[9] Philipp E, Wilckens T, Friess E, Platte P, Pirke KM (1992). Cholecystokinin, gastrin and stress hormone responses in marathon runners. Peptides.

[10] Banfi G, Marinelli M, Bonini P, Gritti I, Roi GS (1996). Pepsinogens and gastrointestinal symptoms in mountain marathon runners. Int J Sports Med.

[11] Garcia-Sancho M, Rodriguez-Franco F, Sainz A, Rodriguez A, Silvan G, Illera JC (2005). Serum gastrin in canine chronic lymphocytic-plasmacytic enteritis. Can Vet J.

[12] Durocher LL, Hinchcliff KW, Williamson KK, McKenzie EC, Holbrook TC, Willard M (2007). Effect of strenuous exercise on urine concentrations of homovanillic acid, cortisol, and vanillylmandelic acid in sled dogs. Am J Vet Res.

[13] Huntingford JL, Levine CB, Mustacich DJ, Corrigan D, Downey RL, Wakshlag JJ (2014). The effects of low intensity endurance activity on various physiological parameters and exercise induced oxidative stress in dogs. Open J Vet Med.

[14] Wakshlag JJ, Stokol T, Geske SM, Greger CE, Angle CT, Gillette RL (2010). Evaluation of exercise-induced changes in concentrations of C-reactive protein and serum biochemical values in sled dogs completing a long-distance endurance race. Am J Vet Res.

[15] Kenyon CL, Basaraba RJ, Bohn AA (2011). Influence of endurance exercise on serum concentrations of iron and acute phase proteins in racing sled dogs. J Am Vet Med Assoc.

[16] Fallon KE (2001). The acute phase response and exercise: the ultramarathon as prototype exercise. Clin J Sport Med.

[17] Sliwowski Z, Lorens K, Konturek SJ, Bielanski W, Zoladz JA (2001). Leptin, gastrointestinal and stress hormones in response to exercise in fasted or fed subjects and before or after blood donation. J Physiol Pharmacol.

[18] McKenzie EC, Jose-Cunilleras E, Hinchcliff KW, Holbrook TC, Royer C, Payton ME (2007). Serum chemistry alterations in Alaskan sled dogs during five successive days of prolonged endurance exercise. J Am Vet Med Assoc.

[19] James FE, Mansfield CS, Steiner JM, Williams DA, Robertson ID (2009). Pancreatic response in healthy dogs fed diets of various fat compositions. Am J Vet Res.

[20] Furr M, Taylor L, Kronfeld D (1994). The effects of exercise training on serum gastrin responses in the horse. Cornell Vet.

[21] Wallace JL, Granger DN (1996). The cellular and molecular basis of gastric mucosal defense. FASEB J.

[22] Markiewicz K, Cholewa M, Lukin M (1979). Gastric basal secretion during exercise and restitution in patients with chronic duodenal ulcer. Acta Hepatogastroenterol (Stuttg).

[23] Feldman M, Nixon JV (1982). Effect of exercise on postprandial gastric secretion and emptying in humans. J Appl Physiol Respir Environ Exerc Physiol.

[24] Kondo T, Naruse S, Hayakawa T, Shibata T (1994). Effect of exercise on gastroduodenal functions in untrained dogs. Int J Sports Med.

[25] Williamson KK, Willard MD, McKenzie EC, Royer CM, Payton ME, Davis MS (2007). Efficacy of famotidine for the prevention of exercise-induced gastritis in racing Alaskan sled dogs. J Vet Intern Med.

[26] Williamson KK, Willard MD, Payton ME, Davis MS (2010). Efficacy of omeprazole versus high-dose famotidine for prevention of exercise-induced gastritis in racing Alaskan sled dogs. J Vet Intern Med.

[27] Davis MS, Willard MD, Nelson SL, McCullough SM, Mandsager RE, Roberts J (2003). Efficacy of omeprazole for the prevention of exercise-induced gastritis in racing Alaskan sled dogs. J Vet Intern Med.

[28] Parente NL (2014). Bari Olivier N, Refsal KR, Johnson CA. Serum concentrations of gastrin after famotidine and omeprazole administration to dogs. J Vet Intern Med.

[29] Snow DH, Rose RJ (1981). Hormonal changes associated with long distance exercise. Equine Vet J.

[30] Angle CT, Wakshlag JJ, Gillette RL, Stokol T, Geske S, Adkins TO (2009). Hematologic, serum biochemical, and cortisol changes associated with anticipation of exercise and short duration high-intensity exercise in sled dogs. Vet Clin Pathol.

[31] Hinchcliff KW, Reinhart GA, Burr JR, Schreier CJ, Swenson RA (1997). Metabolizable energy intake and sustained energy expenditure of Alaskan sled dogs during heavy exertion in the cold. Am J Vet Res.

[32] McConkey B (1984). C reactive protein concentrations during long distance running. Br Med J (Clin Res Ed).

[33] Burr JR, Reinhart GA, Swenson RA, Swaim SE, Vaughn DM, Bradley DM (1997). Serum biochemical values in sled dogs before and after competing in long-distance races. J Am Vet Med Assoc.

[34] Otabe K, Ito T, Sugimoto T, Yamamoto S (2000). C-reactive protein (CRP) measurement in canine serum following experimentally-induced acute gastric mucosal injury. Lab Anim.

[35] Mordecai A, Sellon RK, Mealey KL (2011). Normal dogs treated with famotidine for 14 days have only transient increases in serum gastrin concentrations. J Vet Intern Med.

[36] Behrend EN, Kemppainen RJ, Young DW (1998). Effect of storage conditions on cortisol, total thyroxine, and free thyroxine concentrations in serum and plasma of dogs. J Am Vet Med Assoc.

[37] Doumatey AP, Zhou J, Adeyemo A, Rotimi C (2014). High sensitivity C-reactive protein (Hs-CRP) remains highly stable in long-term archived human serum. Clin Biochem.

[38] Li Q, Kang T, Tian X, Ma Y, Li M, Richards J (2013). Multimeric stability of human C-reactive protein in archived specimens. PLoS One.

